# Positive and Negative Regulators of Sclerostin Expression

**DOI:** 10.3390/ijms23094895

**Published:** 2022-04-28

**Authors:** Rina Iwamoto, Masanori Koide, Nobuyuki Udagawa, Yasuhiro Kobayashi

**Affiliations:** 1 Division of Hard Tissue Research, Institute for Oral Science, Matsumoto Dental University, 1780 Gobara Hiro-oka, Shiojiri 399-0781, Nagano, Japan; rina.iwamoto@mdu.ac.jp (R.I.); masanori.koide@mdu.ac.jp (M.K.); 2 Department of Biochemistry, Matsumoto Dental University, 1780 Gobara Hiro-oka, Shiojiri 399-0781, Nagano, Japan; nobuyuki.udagawa@mdu.ac.jp

**Keywords:** sclerostin, Wnt inhibitor, bone remodeling, osteoclast, osteocyte, LIF

## Abstract

Sclerostin is secreted from osteocytes, binds to the Wnt co-receptor Lrp5/6, and affects the interaction between Wnt ligands and Lrp5/6, which inhibits Wnt/β-catenin signals and suppresses bone formation. Sclerostin plays an important role in the preservation of bone mass by functioning as a negative regulator of bone formation. A sclerostin deficiency causes sclerosteosis, which is characterized by an excess bone mass with enhanced bone formation in humans and mice. The expression of sclerostin is positively and negatively regulated by many factors, which also govern bone metabolism. Positive and negative regulators of sclerostin expression and their effects are introduced and discussed herein based on recent and previous findings, including our research.

## 1. Introduction

Bone tissues repeat a cycle of bone resorption and formation throughout their life after reaching their peak bone mass [1,2,3,4]. This process, called bone remodeling, is performed to preserve bone mass and maintain calcium homeostasis. Osteoclasts, multinucleated bone-resorbing cells, differentiate from monocyte/macrophage progenitor cells [5]. Their differentiation is tightly regulated by osteoblast-lineage cells, such as osteoblasts and osteocytes [6,7]. Osteoblasts constitutively express macrophage colony-stimulating factors (M-CSF; encoded by the *Csf1* gene), an essential cytokine for the differentiation and survival of macrophage-lineage cells, including osteoclasts, and osteoblasts and osteocytes inducibly express a receptor activator of the NF-κB ligand (RANKL; encoded by the *Tnfsf11* gene), an essential cytokine for osteoclastogenesis, when stimulated with bone-resorbing factors [8,9]. Osteoprotegerin (OPG; encoded by the *Tnfrsf11b* gene) is also expressed by osteoblast-lineage cells to inhibit osteoclastogenesis [10]. When M-CSF binds to its receptor Csf1r, and RANKL binds to receptor activator of NF-κB (RANK; encoded by the *Tnfrsf11a* gene) in osteoclast precursors, the differentiation of osteoclasts is initiated. Mature osteoclasts adhere to the bone surface and then secrete hydrogen chloride and several proteases, including cathepsin K, into bone resorption lacunae for the resorption of mineralized matrices and matrix proteins, such as type I collagens [11].

After the completion of osteoclastic bone resorption, osteoblasts secrete and add new bone matrices to resorption lacunae. Some osteoblasts are embedded in the newly formed matrix and differentiate into osteocytes [12]. Osteocytes communicate with each other or with osteoblasts and osteoclasts through their dendrites to sense mechanical stimuli transmitted in bone and then regulate bone remodeling. Recent studies revealed that osteocytes function as an endocrine organ to actively regulate bone mineral metabolism by secreting the phosphate-metabolic hormone Fibroblast growth factor 23 as well as RANKL and OPG [13,14]. Osteocytes also secrete Wnt1 and the Wnt inhibitor sclerostin to tightly regulate bone mass [15,16].

Cytokine Wnt ligands are involved in the development of organs and the maintenance of stem cells in various tissues [17]. In 2001, low-density lipoprotein receptor-related protein 5 (LRP5) was identified as a causative gene for osteoporosis pseudoglioma syndrome (OMIM: 258770) and functions as a Wnt co-receptor to promote bone accrual by enhancing Wnt/β-catenin signals [18]. *Sost* was also identified as a causative gene for sclerosteosis (OMIM: 269500) and Van Buchem disease (OMIM: 239100) [19,20,21]. Since then, a large number of studies have reported the roles of Wnt ligands and Wnt inhibitors in bone metabolism in addition to the successful application of sclerostin antibodies to the treatment of osteoporosis. In this review, we focus on positive and negative regulators of sclerostin expression and discuss previous and recent findings, including our research, the mechanisms by which sclerostin is expressed in osteocytes, and how these factors regulate its expression.

## 2. The Canonical Wnt Signaling and Its Role in Bone Formation

Wnt signals induce various biological events through β-catenin-dependent canonical and -independent non-canonical pathways [17]. In the absence of Wnt ligands, β-catenin is phosphorylated by the β-catenin destruction complex of Axin2, adenomatous polyposis polyps, and glycogen synthase kinase-3β, and phosphorylated β-catenin is then ubiquitinated and degraded by proteasomes. Once Wnt ligands bind to their receptor complex of Frizzled and the co-receptor LRP5/6, the activity of the β-catenin destruction complex is suppressed and β-catenin accumulates in the cytosol. Accumulated β-catenin is translocated into the nucleus and promotes the expression of its target genes together with T-cell factor (TCF)/lymphocyte enhancer factor 1.

Limb bud mesenchymal cell-selective (Prrx1-Cre; *Wnt1*^fl/fl^) and osteoblast-lineage cell-selective (Osterix-Cre; *Wnt1*^fl/fl^ or Dmp1-Cre; *Wnt1*^fl/fl^) *Wnt1*-deficient mice exhibited a low bone mass phenotype [22,23,24]. Furthermore, the overexpression of Wnt1 promoted the nuclear translocation of β-catenin in the murine embryo fibroblast cell line C3H10T1/2 [22]. The decreased expression of target genes for the canonical Wnt pathways was detected in bone tissues from Osterix-Cre; *Wnt1*^fl/fl^ mice [23]. Collectively, these findings indicate that Wnt1 promotes bone formation via the canonical Wnt pathway. Furthermore, the osteoblast-lineage cell-specific expression of Wnt1 increased bone mass. The enhancing effects of Wnt1 on bone mass were not cancelled by the loss of Lrp5, even though *Lrp5*-deficient mice exhibited a low bone mass [24]. This finding suggests that Wnt1 positively regulated bone mass in a Lrp5-independent manner. Moreover, Cawthorn et al. reported that the overexpression of Wnt6, Wnt10a, and Wnt10b promoted osteoblast differentiation in the bone marrow stromal cell line ST2 and suppressed adipocyte differentiation in cultures of the preadipocyte cell line 3T3-L1 [25]. The knockdown of β-catenin in these cells impaired Wnt6-, Wnt10a-, and Wnt10b-induced osteoblastogenesis and promoted adipogenesis [25]. Therefore, the activation of canonical Wnt pathways in osteoblast-lineage cells drives anabolic effects on bone. Further studies are needed to clarify which Wnt ligands are important for the promotion of bone formation in vivo.

## 3. Mechanism of Action of Sclerostin

Sclerostin (encoded by the *Sost* gene) binds to the Wnt co-receptor LRP5/6 and inhibits Wnt/β-catenin signals. A previous study using nuclear magnetic resonance spectroscopy showed the three-dimensional structure of sclerostin [26]. The long N- and C-terminal regions of sclerostin are unstructured and highly flexible. The remaining region forms three loop structures with a cysteine knot. The extracellular domain of LRP5/6 consists of four β-propeller/epidermal growth factor repeats and three low-density lipoprotein (LDL) repeats. Holdsworth et al. demonstrated that the NXI (Asn-X-Ile) motif in the loop2 region of sclerostin was important for binding to the E1 domain of LRP6 [27]. Kim et al. recently showed that the affinity of sclerostin was higher for LRP6 containing the E1-E2 domains than for that containing E1 only [28]. This finding indicates that sclerostin binds to the E2 domain of LRP6 as well as its E1 domain. Furthermore, the C-terminal region of sclerostin was shown for the first time to bind to the E2 domain of LRP6. Therefore, the loop2 and c-terminal regions of sclerostin interact with the E1 and E2 domains of LRP5/6, respectively.

Bourhis et al. investigated the mechanisms by which Wnt ligands bind to LRP5/6 and showed that Wnt3a interacted with the E3 and E4 domains of LRP6 and that Wnt9b interacted with its E1 and E2 domains [29]. Therefore, Wnt ligands bind to their binding domains of LRP5/6, which differ depending on each ligand.

Since sclerostin binds to the E1-E2 domains of LRP6, it may inhibit Wnt signals induced by Wnt1, Wnt2, and Wnt9a, which bind to E1-E2 of LRP6. In contrast, sclerostin cannot inhibit Wnt3a-induced Wnt signals. Furthermore, the loop2, but not c-terminal, region of sclerostin is important for its inhibitory effects on Wnt1. In contrast, the c-terminal region of sclerostin is more important for its inhibitory effects on Wnt2 and Wnt9b. Dkk1 was shown to bind to the E1 and E3 domains of LRP6 and inhibited Wnt1, Wnt2, Wnt9b, and Wnt3a [28,30]. Therefore, structure–functional interactions among Wnt ligands, receptors, and Lrp5/6 have been clarified.

LRP4 is required for the binding of sclerostin to LRP5/6. Patients with mutations (R1170W, W1186S) in the *Lrp4* gene exhibited a high bone mass, similar to patients with sclerosteosis [31,32]. These mutations were detected in the central domain of the third propeller of LRP4, which is needed for the interaction with sclerostin. Mice, in which the mutation (R1170W) was introduced into the *Lrp4* gene using a CRISPR/Cas9 approach, exhibited a high bone mass [33]. Serum sclerostin levels were higher in osteoblast-lineage cell-selective *Lrp4*-deficient mice. This finding suggests that Lrp4 retains sclerostin in bone tissues. Therefore, LRP4 appears to facilitate the binding of sclerostin to LRP5/6 in the microenvironment of bone tissues.

## 4. Roles of Sclerostin in Bone Development and Bone Formation

Sclerostin was initially considered to be an inhibitor of bone morphogenetic protein (BMP) because of its homology to the differential screening-selected gene in neuroblastoma family members, such as the BMP antagonist noggin. Immunoprecipitation assays showed that sclerostin bound to BMP6 and that this binding was competitively inhibited by the BMP receptor BMPRIA. Sclerostin mildly suppressed the phosphorylation of SMAD by BMP6 and the mineralization of human mesenchymal cells. *Sost* transgenic mice had a low bone mass with impaired bone formation [34]. Sclerostin was subsequently shown to bind to LRP5/6 and suppress Wnt/β-catenin signaling [35].

Four-month-old *Sost*-deficient (*Sost* KO) mice showed a high bone mass phenotype with increased bone formation and unchanged bone resorption [36,37]. Bone mineral density was significantly higher in *Sost* KO mice at 4 weeks of age, and the difference in bone mineral density between wild-type and *Sost* KO mice increased with aging. In addition, there were no gross abnormalities in the tibia at E14.5 or in the head and forelimbs on postnatal day 1. We also generated *Sost* KO mice, examined their bone phenotype [38], and found that bone mass was normal in 6-week-old *Sost* KO mice (unpublished data). These findings indicate that sclerostin is not involved in bone development. The percentage of sclerostin-positive osteocytes in bone was reported to be lower in 3-day-old mice than in 4-week-old mice [39]. The percentage of sclerostin-positive osteocytes also increased with aging [40]. Collectively, these findings indicate that sclerostin does not play an important role in skeletal development, which may be due to its low expression during the developmental process.

## 5. Roles of Sclerostin in Bone Resorption

Mice, in which the constitutively active form of β-catenin was expressed in osteoblasts, exhibited an osteopetrotic phenotype with impaired osteoclastogenesis due to the increased expression of OPG [41]. The proximal 3.6 kb *OPG* promoter assay revealed that TCF proteins, together with β-catenin, induced the expression of OPG in osteoblasts. Furthermore, osteoblast-selective β-catenin conditional knockout mice had a markedly decreased bone mass with an increased number of osteoclasts on the endocortical surfaces due to the decreased expression of OPG in osteoblasts and osteocytes [41]. These findings suggest that the activation of Wnt/β-catenin signals in osteoblasts induces the expression of OPG, which, in turn, suppresses osteoclast differentiation.

Although the activation of Wnt/β-catenin signals suppresses bone resorption, serum levels of bone resorption markers, such as TRAP5b, and histomorphometric markers of osteoclast surface/bone surface were normal in 4-month-old *Sost* KO mice [36,37]. In contrast, bone resorption was reportedly impaired in *Lrp4* cKO mice. Chang et al. reported mild decreases in the number of osteoclasts in 3-month-old male, but not female, osteocalcin (Ocn)-cre; *Lrp4*^fl/fl^ mice [32]. Furthermore, Xiong et al. showed that the serum level of the bone resorption marker deoxypyridinoline and the number of osteoclasts were markedly lower in 3-month-old Ocn-Cre; *Lrp4*^fl/fl^ mice [42]. A treatment with sclerostin increased RANKL expression and decreased OPG expression in bone marrow stromal cell cultures prepared from wild-type mice, but not those from *Lrp4* KO mice. Sclerostin also up-regulated RANKL expression in human osteoblast-like cells and mouse osteocyte-like MLO-Y4 cells [43]. These findings indicate that the binding of sclerostin to Lrp4 increased the expression of RANKL and decreased the expression of OPG. However, this model does not explain why bone resorption was suppressed in *Lrp4* KO mice, but not in *Sost* KO mice. Therefore, the binding of unidentified ligands to Lrp4 may enhance RANKL expression.

## 6. The Structure of the *Sost* Gene

Loss-of-function mutations in the *Sost* gene cause sclerosteosis (OMIM: 269500) and Van Buchem disease (OMIM: 239100), which are both characterized by a high bone mass. Three mutations have been identified in the *Sost* gene of patients with sclerosteosis: the C69T mutation in the first exon and two splicing mutations [19]. Baleman et al. reported one splicing mutation and two nonsense mutations in the second exon [20]. In patients with Van Buchem disease, no mutations were found in the coding region of the *Sost* gene, whereas a 52 kb deletion was identified between the *Sost* and *MEOX1* genes. This deletion started 35 kb downstream of the *Sost* gene [21].

**Figure 1:**
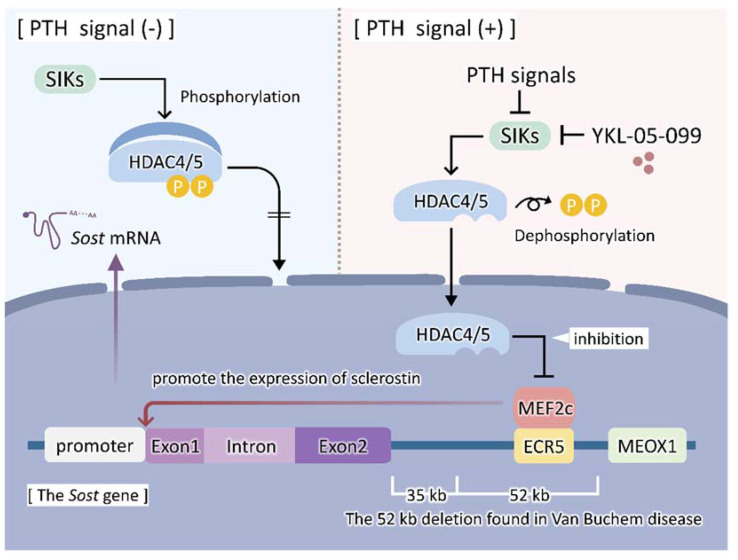
Structure of the *Sost* gene and effects of PTH on the expression of sclerostin. PTH signals suppress the expression of sclerostin. In the absence of PTH signals, salt-inducible kinase (SIK) 2 phosphorylates HDAC4/5 to promote the cytoplasmic retention of HDAC4/5. MEF2c binds to ECR5 (enhancer region) of the *Sost* gene to promote the expression of sclerostin. PTH signals inhibit the phosphorylation of HDAC4/5 by SIK2. Dephosphorylated HDAC4/5 remain in the nucleus and inhibit the transcriptional activity of MEF2c. YKL-05-099, a small-molecule SIK inhibitor, mimics the effects of PTH. PTH, parathyroid hormone; SIK, salt-inducible kinase; HDAC, histone deacetylase; MEF, myocyte enhancer factor; ECR, evolutionarily conserved region; MEOX1, mesenchyme homeobox 1.

Mice expressing the wild-type *Sost* gene exhibited a low bone mass, whereas those expressing the *Sost* gene lacking the 52 kb region lost in Van Buchem disease did not. Furthermore, ECR5 was important for the bone-specific expression of sclerostin among the seven regions conserved in the 52 kb region of the human and mouse *Sost* gene. These findings revealed that the ECR5 region is required for the expression of sclerostin in bone [44]. The Mef-binding motif was detected in the ECR5 region based on the inhibitory effects of parathyroid hormone (PTH) on sclerostin expression. Among Mef family members, the expression of Mef2c was the highest in bone, suggesting that it was recruited into ECR5, which, in turn, induced the expression of sclerostin. Osteoblast and osteocyte-specific *Mef2c* cKO mice or osteoblast and osteocyte-specific deletion of ECR5 mice showed a high bone mass, similar to *Sost* KO mice [45]. Therefore, Mef2c binds to the bone tissue-specific enhancer ECR5 and induces the expression of sclerostin in osteocytes (Figure 1).

## 7. Regulation of Sclerostin Expression

### 7.1. PTH

The anabolic effects of PTH on bone are exerted in part by inhibiting the expression of sclerostin [46]. Since HDAC family members reportedly suppress the transcriptional activity of Mef2, the role of HDAC in the inhibitory effects of PTH on sclerostin expression was investigated [47,48]. The overexpression of HDAC5 suppressed the activity of the *Sost* enhancer in UMR106 cells, which are known to express sclerostin. Moreover, the knockdown of HDAC5 promoted the transcriptional activity of Mef2c in osteocyte-like cells. The percentage of sclerostin-positive osteocytes increased in *Hdac5* KO mice. Wein et al. showed that the knockdown of Class II HDAC HDAC4/5 cancelled the inhibitory effects of PTH on sclerostin expression and also that salt-inducible kinase (SIK) 2 mediated the PTH-induced suppression of HDAC4/5 activity [49]. In the absence of PTH signals, SIK phosphorylates HDACs, which results in their cytosolic retention. PTH signals inhibit SIK2 and then suppress the phosphorylation of HDAC4/5. Dephosphorylated HDACs are retained in the nucleus, suppress the transcriptional activity of Mef2c, and then inhibit the expression of sclerostin (Figure 1). The SIK2 inhibitors YKL-05-093 and -099 have been developed, and the administration of YKL-05-099 increased bone mass with enhanced bone formation in 8-week-old male mice. In addition, sclerostin was more recently reported to be degraded in lysosomes. A stimulation with fluid shear stress and PTH degraded the sclerostin protein in bone, osteocyte-like cells, and UMR106 cells within 30 min [50]. This finding suggests that the rapid degradation of sclerostin in osteocytes is also important for decreasing sclerostin proteins as soon as possible. Further studies are needed to clarify the importance of the rapid lysosomal degradation of sclerostin for the bone anabolic effects of FSS and PTH in vivo.

### 7.2. PGE_2_

Prostaglandin E_2_ (PGE_2_) binds to EPs1–4 receptors. EP2 and EP4 are coupled to the Gs protein and activate adenylate cyclase-cAMP-PKA signals. EP1 is coupled to Gq-phospholipase C. EP3 is coupled to Gi and inhibits adenylate cyclase [51]. Treatments with PGE_2_ have been shown to suppress sclerostin expression in UMR106 cells [52,53,54]. The siRNA-mediated knockdown of EP2 and EP4 and treatments with receptor-specific agonists revealed that EP2 mediated the suppressive effects of PGE_2_ on sclerostin expression in UMR106 cells. A treatment with PGE_2_ was previously reported to decrease sclerostin expression, even in UMR106 cells, in which the expression of Mef2c was suppressed by siRNA, suggesting that the effects of PGE_2_ were not mediated by Mef2c [52]. However, PGE_2_ may inhibit the residual activity of Mef2c to suppress sclerostin expression due to the incomplete knockdown of Mef2c. Therefore, further studies are needed to clarify the roles of PGE_2_ in sclerostin expression in bone metabolic diseases, including osteoporosis.

### 7.3. Mechanical Loading

Mechanical loading promotes bone formation and increases bone mass. Osteocytes sense mechanical loading transmitted to bone and down-regulate the expression of sclerostin [55,56]. In contrast, the expression of sclerostin was increased in the unloaded tibiae of tail suspension model mice [56]. These findings indicate that mechanical loading decreases the expression of sclerostin, which, in turn, promotes bone formation.

Previous studies proposed a mechanism by which mechanical loading increases bone mass [57,58]. Since the Ca channel piezo is activated by mechanical stimuli in several cells, the bone phenotypes of osteoblast-specific (Osteocalcin-Cre; *Piezo1*^fl/fl^) and osteoblast/osteocyte-specific (Dmp1-Cre; *Piezo1*^fl/fl^) *Piezo1* cKO were investigated [59,60]. These mice exhibited a low bone mass with impaired bone formation and the mild enhancement of bone resorption. The stimulation with mechanical loading increased the expression of osteoblast marker genes, such as *Alpl* (encoding alkaline phosphatase) and *Bglap* (encoding osteocalcin), in control osteoblasts, but not in *Piezo1* cKO osteoblasts (Osteocalcin-Cre; *Piezo1*^fl/fl^) [59]. The expression of *Wnt1* mRNA was decreased in 5-week-old Dmp1-Cre; *Piezo1*^fl/fl^ mice [60]. However, the expression of sclerostin remained unchanged. The treatment of the osteocyte-like cell line MLO-Y4 with the Piezo1 agonist Yoda1 decreased the expression of sclerostin and increased that of Wnt1. Furthermore, mechanical loading decreased sclerostin expression in osteocyte-like IDG-SW3 cells. In contrast, the inhibitory effects of mechanical loading on sclerostin expression were abrogated by the treatment with the Piezo1 inhibitor GsMTx4 and Akt inhibitor IV as well as the knockout of the *Piezo1* gene in these cells [61]. These findings suggest that Piezo1 activates Akt signals and then suppresses the expression of sclerostin. The mechanisms by which Akt signals suppress sclerostin expression and the reason why the deletion of Piezo1 had no effect on sclerostin expression in vivo currently remain unclear. PGE_2_ is reportedly involved in the suppressive effects of mechanical loading on sclerostin expression [52]. Collectively, these findings indicate that Piezo1 signals cooperate with PGE_2_ signals to suppress sclerostin expression in vivo.

### 7.4. Gp130 and IL-6 Family Cytokines

Leukemia inhibitory factor (LIF), oncostatin M (OSM), and cardiotrophin-1 (CT-1) were previously shown to inhibit the expression of sclerostin in UMR106 cells [62]. OSM was highly expressed in osteoblasts, suggesting that OSM secreted from osteoblasts functions as a positive regulator of bone mass to suppress sclerostin expression and promote bone formation. CT-1 expression was detected in osteoclasts and enhanced bone formation [63]. Therefore, CT-1 suppresses sclerostin expression and plays an important role in the transition from bone resorption to formation during bone remodeling. However, the importance of LIF in the suppression of sclerostin expression has not yet been elucidated.

**Figure 2:**
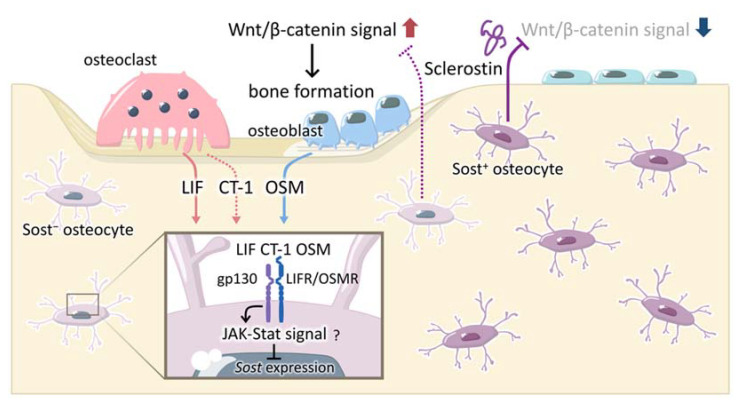
Roles of LIF, CT-1, and OSM in bone remodeling. Osteoclast-derived LIF, CT-1, and osteoblast-derived OSM inhibit the expression of sclerostin in osteocytes. These cytokines bind to gp130 and LIFR/OSMR, and may inhibit *Sost* expression via the JAK-Stat signal. The inhibition of sclerostin expression enhances Wnt/β-catenin signals in osteoblasts, which, in turn, promote bone formation. LIF, leukemia inhibitory factor; CT-1, cardiotropin-1; OSM, oncostatin M; LIFR, LIF receptor; OSMR, OSM receptor; JAK-Stat, janus kinase-signal transducer and activator of transcription.

OPG KO mice exhibited a low bone mass with enhanced bone resorption and formation [64]. The expression of Axin2, a target gene of Wnt/β-catenin signals, and the number of β-catenin-positive osteoblasts were increased in bone tissues from these mice, suggesting the enhancement of Wnt/β-catenin signals in OPG KO mice [54]. An increase in the expression of any Wnt ligand has not yet been detected in OPG KO mice. In contrast, the expression of sclerostin was markedly decreased in these mice, suggesting that enhanced bone resorption or osteoclasts themselves suppress sclerostin expression. The treatment of UMR106 cells with conditioned medium (CM) prepared from osteoclast cultures inhibited sclerostin expression. Using protein arrays, LIF was proposed as a candidate factor that suppresses sclerostin expression. A real-time PCR analysis and immunohistochemical studies revealed that LIF was highly expressed in osteoclasts. A recent study using RNA sequencing and gene expression analyses also showed that LIF was highly expressed in human osteoclasts in vitro, but not in human osteocyte- and osteoblast-enriched fractions prepared from bone biopsies [65]. This finding also indicates that LIF is highly expressed in human osteoclasts as well as in mouse osteoclasts. The administration of an anti-RANKL antibody completely inhibited osteoclast formation in OPG KO mice, which resulted in the complete disappearance of LIF-positive osteoclasts and increases in sclerostin-positive osteocytes. Anti-LIF neutralizing antibodies, but not anti-OSM or anti-CT-1 neutralizing antibodies, cancelled the inhibitory effects of osteoclast CM on sclerostin expression in UMR106 cell cultures. These findings indicate that LIF secreted from osteoclasts acts in osteocytes and then suppresses sclerostin expression, which, in turn, promotes bone formation. Therefore, LIF secreted from osteoclasts may act in osteocytes as a coupling factor between bone resorption and formation (Figure 2).

To clearly observe cells expressing sclerostin, *Sost* ^Green/+^ mice, in which a part of exon 1 after the *Sost* start codon was replaced with ZsGreen cDNA (coding green fluorescent protein), were generated [38]. The number of ZsGreen-positive osteocytes was lower in trabecular bone than in cortical bone. In contrast, the number of osteoclasts was higher in trabecular bone. When anti-RANKL antibodies were injected into wild-type mice, the number of LIF-positive osteoclasts in trabecular bone markedly decreased, whereas the number of sclerostin-positive osteocytes increased. This finding suggests that osteoclast-derived LIF suppresses sclerostin expression, thereby promoting bone remodeling. Gp130 and IL-6 family cytokines are known to activate JAK-Stat signals. Therefore, further studies are needed to clarify how these cytokines suppress sclerostin expression through these signals.

### 7.5. 1α,25(OH)_2_D_3_

Chromatin immunoprecipitation coupled with high-throughput sequencing showed that 1α,25(OH)_2_D_3_ suppressed sclerostin expression; however, the binding of vitamin D receptors (VDR) was not observed adjacent to the *Sost* gene in IDG-SW3 cell cultures [66]. A mini gene assay showed that the deletion of the ECR5 region and mutations in Mef2-binding sites on the *Sost* gene abrogated the suppressive effects of the PKA activator forskolin on sclerostin expression, but not those of 1α,25(OH)_2_D_3_. The deletion of a 1 kb proximal promoter of the *Sost* gene also did not alter the effects of 1α,25(OH)_2_D_3_. These findings suggest that 1α,25(OH)_2_D_3_ suppresses sclerostin expression in Mef2c- and proximal promoter-independent manners.

A real-time PCR analysis showed that sclerostin expression levels in bone were higher in osteoblast-lineage cell-selective VDR cKO mice than in control mice [67]. The administration of the vitamin D_3_ analog eldecalcitol increased sclerostin expression in bone tissues from control mice, but not those from VDR cKO [67]. These findings indicate that endogenous 1α,25(OH)_2_D_3_ suppresses sclerostin expression via VDR and that a pharmacological dose of vitamin D_3_ enhances sclerostin expression. Wijenayaka et al. reported that 1α,25(OH)_2_D_3_ increased sclerostin expression in human primary osteoblasts [68]. They also demonstrated that a VDR-responsive element at a position -6216 bp upstream of the transcription start site on the human *Sost* gene was essential for 1α,25(OH)_2_D_3_ responsiveness [68]. Therefore, the effects of 1α,25(OH)_2_D_3_ on sclerostin expression may differ between humans and mice. Furthermore, there may be a difference in the mechanisms of action of endogenous and exogenous 1α,25(OH)_2_D_3_, including its analogs.

## 8. Positive Regulators of Sclerostin Expression

A previous study reported that BMP2 increased the expression of the transcriptional factor osterix and sclerostin in primary osteoblasts [69]. An in situ hybridization analysis showed that osterix-positive areas overlapped with sclerostin-positive areas in embryonic calvariae and limb buds, suggesting that BMP2 enhanced sclerostin expression by inducing the expression of osterix [69]. An analysis of osteoblast-lineage cell-selective Bmpr1a cKO mice revealed that BMP signals enhanced the expression of sclerostin and Dkk1 through Smad- and p38 mitogen-activated protein kinase (p38MAPK)-dependent signals, respectively [70]. Osteoblast-lineage cell-selective Bmpr1a cKO mice exhibited a high bone mass with increased bone formation due to the suppression of sclerostin and Dkk1 expression. Since Smad-binding sites have not yet been identified adjacent to the *Sost* gene, it currently remains unclear whether BMP2-Smad signals directly regulate sclerostin expression.

Dkk1 is a target gene for Wnt/β-catenin signals. The administration of anti-sclerostin antibodies has been shown to enhance Wnt/β-catenin signals, which increased the expression of Dkk1 [71]. In contrast, the administration of anti-Dkk1 antibodies increased the expression of sclerostin [72], suggesting that sclerostin and Dkk1 are target genes for Wnt/β-catenin signals. Therefore, the expression of sclerostin is positively regulated by BMP and Wnt signals.

After bone mass plateaus at approximately 20 years of age, it gradually decreases with aging. Aged humans and animals exhibited a low turnover in bone metabolism, with significant decreases in both bone resorption and formation. Previous studies investigated whether changes in sclerostin expression were associated with a low bone mass with aging. Serum sclerostin levels were significantly higher in men than in women and positively correlated with age in both groups [73]. Other studies demonstrated that serum sclerostin levels were higher in aged men and women [74,75]. Roforth et al. reported that the mRNA expression of *Sost* did not significantly differ between young (mean age, 30.0 years) and old (mean age, 72.9 years) women [75]. Serum sclerostin levels correlated with total hip bone mineral density; however, higher serum sclerostin levels were associated with a greater risk of hip fractures in older women [76]. In contrast, serum sclerostin levels were associated with a low risk of fractures, low bone mineral density, and low turnover of bone metabolism [77]. The mRNA expression of *Sost* in bone tissues was higher in 6-week-old mice than in 6- and 18-month-old mice [78]. Furthermore, the depletion of senescent cells reportedly reduced the mRNA expression of *Sost* in aged mice [79]. Therefore, there is currently no consensus on the mRNA expression of *Sost* in humans or mice. These findings indicate that aging is not a major factor regulating sclerostin expression in osteocytes. Aging may also affect the positive and negative regulators of sclerostin expression described above, thereby intricately regulating sclerostin expression in aged bone tissues.

## 9. Role of Sclerostin in Rheumatoid Arthritis

Sclerostin is certainly involved in the onset of osteoporosis because the administration of anti-sclerostin antibodies (Romosozumab) increased bone formation and decreased bone resorption, which, in turn, markedly increased bone mass in osteoporosis models and patients as previously reported [80,81,82,83,84]. In addition to this, several studies reported that the effects of sclerostin antibodies on bone loss were associated with inflammatory bone diseases such as rheumatoid arthritis. The administration of sclerostin antibody reportedly prevented the decreased systemic bone mineral density, but not focal inflammations such as joint swelling and synovitis, or the focal bone erosions [85,86]. These findings indicate that the sclerostin inhibition effectively increases systemic bone mass even under inflammatory conditions. Furthermore, Wehmeyer et al. reported that sclerostin was expressed in synovial tissues from rheumatoid arthritis patients, and the paw swelling and joint destruction were exacerbated in human tumor necrosis factor-α (TNF) transgenic arthritis models when the *Sost* gene was targeted in those mice [87]. Sclerostin inhibited TNF-induced activation of p38MAPK in fibroblast-like synoviocytes. The expressions of *Sost* and *Dkk1* were transiently increased before the onset of arthritis in adjuvant-induced arthritis models, suggesting that sclerostin expression is associated with the onset of arthritis as well as Dkk1 [88,89]. Thus, in addition to the inhibitor of Wnt/β-catenin signals, sclerostin may have a role in the suppression of TNF-induced inflammation in rheumatoid arthritis.

## 10. Role of Sclerostin in Bone Cancers Including Multiple Myeloma

Sclerostin is involved in bone loss associated with multiple myeloma and bone cancers and possibly involved in their pathogenesis [90]. The application of anti-sclerostin antibodies is expected for the treatments of bone cancers. Clinical studies reported that serum levels of sclerostin positively correlate with the progression of multiple myeloma as well as those of macrophage inflammatory protein-1α which is secreted from multiple myeloma plasma cells and involved in the development of myeloma-related bone diseases [90,91,92]. Eda et al. investigated sources of sclerostin and efficiencies of treatments with anti-sclerostin antibodies with a proteasome inhibitor Carfilzomib approved for the treatment of multiple myeloma using humanized multiple myeloma xenograft model mice [92]. Multiple myeloma cells reportedly stimulated the expression of sclerostin in osteoblasts through Dkk1. An immunohistochemical study showed that myeloma cells were markedly decreased in these mice treated with anti-sclerostin antibodies and Carfilzomib, but not treated with only anti-sclerostin antibodies or Carfilzomib. Sclerostin also secreted from myeloma cells and inhibited the differentiation of human bone marrow stroma cells into osteoblasts in cultures [93]. In contrast, MacDonald et al. showed that the expression of sclerostin was not detected in plasma cells from 630 myeloma patients or 54 myeloma cell lines and that the anti-sclerostin antibody failed to reduce tumor burden [94]. Thus, roles of sclerostin in the development of multiple myeloma have been controversial.

Breast cancer cells in bone metastasis regions reportedly expressed sclerostin, and treatment of breast cancer cells with anti-sclerostin antibodies inhibited the proliferation and migration of these cells in cultures [95]. However, treatment with anti-sclerostin antibodies failed to suppress tumor growth in xenograft model mice, in which breast cancer cells were injected into their bone marrows. Hesse et al. reported that sclerostin secreted from breast cancer cells induced muscle fiber atrophy by enhancing NF-κB and p38MAPK signals as well as enhancements of bone destructions by the inhibition of Wnt/β-catenin signals in osteoblasts [96]. Anti-sclerostin antibodies reduced bone metastasis of breast cancer cells with the suppression of bone destructions. They also decreased muscle fiber atrophy by partially restoring pax7-positive satellite cells. Mechanistically, the cancer-induced muscle fiber atrophy was mediated by bone matrix-derived transforming growth factor-β released by the enhanced bone resorption. These findings suggest that anti-sclerostin antibodies are effective not only the prevention of bone destructions, but also the improvement of muscle functions impaired by breast cancer metastasis. Further studies are needed to clarify the mechanisms by which ectopic expression of sclerostin occurs in multiple myeloma and bone metastasis of breast cancers.

## 11. Conclusions

In this review, we focused on positive and negative regulators of sclerostin expression in osteocytes and discussed the molecular mechanisms by which they regulate sclerostin expression (see Table 1). Among them, the suppressive effects of PTH on sclerostin expression have been intensively investigated. The findings obtained led to the development of small-molecule SIK2 inhibitors that suppress sclerostin expression, thereby increasing bone mass. These SIK2 inhibitors may replace anti-sclerostin antibodies in the treatment of osteoporosis in the future. However, the regulatory mechanisms underlying sclerostin expression by most of these factors, except for PTH, have not yet been elucidated at the molecular level. These mechanisms will be clarified in the future, and new drugs that suppress sclerostin expression will be developed for the treatment of bone metabolic diseases.

## Author Contributions

R.I. and Y.K. wrote the manuscript. R.I. prepared the figures. All authors (R.I., M.K., N.U. and Y.K.) discussed the interpretation of previous findings introduced in the manuscript. All authors have read and agreed to the published version of the manuscript.

## Figures and Tables

**Table 1:** Regulators of sclerostin expression.

Negative Regulator	Cell Line or Animal Models	*Sost* Exp/Sclerostin	Refs
PTH	Col1-Cre; *Mef2c*^fl/fl^	↓	[45]
	UMR106 cell	↓	[46,48]
	*Hdac5* KO	↓	[47]
	Ocy454 cell	↓	[47,50]
	Tibiae from PTH-treated mice	↓	[49]
	Dmp1-Cre; *Hdac4*^fl/fl^; *Hdac5*^−/−^	↓	[49]
PGE_2_	UMR106 cell	↓	[52,53,54]
Mechanical loading	UMR106 cell with fluid shear stress	↓	[50]
	Saos2 cell with mechanical strain	↓	[52]
	IDG-SW3 cell with mechanical strain	↓	[61]
gp130 and IL-6 family cytokines	UMR106 cell treated with LIF	↓	[54]
	calvarial osteoblasts treated with OSM	↓	[62]
	UMR106 cell treated with LIF, OSM, CT-1	↓	[62]
1α,25(OH)_2_D_3_	IDG-SW3 cell	↓	[66]
	Osterix-Cre; *VDR*^fl/fl^ mice	↓	[67]
	long bones from Eldecalcitol-treated mice	↑	[67]
	human primary osteoblast	↑	[68]
**Positive Regulator**	**Cell Line or Animal Models**	***Sost* Exp/Sclerostin**	**Refs**
BMP	Col1-CreER; *Bmpr1a*^fl/fl^	↑	[70]
Wnt	Administration of anti-DKK1 antibody	↑	[72]
Aging	human serum	↑	[74,75]
	human bone samples	→	[75]
	Mouse bone	↑	[78]
	depletion of senescence cells	↑	[79]

## Data Availability

No new data were created and analyzed in this study. Data sharing is not applicable to this article.
